# Corticosteroid Injection Versus Surgical Decompression in Carpal Tunnel Syndrome: A Prospective Comparative Interventional Study Using Clinical, Electrophysiological, and Sonographic Outcomes

**DOI:** 10.7759/cureus.113881

**Published:** 2026-08-03

**Authors:** Shubhi Shukla, Anurag Agarwal, Anjali Singh, Shivani Rastogi, Swagat Mahapatra, Shamrendra Narayan, Samiksha Parashar

**Affiliations:** 1 Anaesthesiology, Dr. Ram Manohar Lohia Institute of Medical Sciences, Lucknow, IND; 2 Anaesthesia, Critical Care and Pain Medicine, Dr. Ram Manohar Lohia Institute of Medical Sciences, Lucknow, IND; 3 Orthopedic Surgery, Dr. Ram Manohar Lohia Institute of Medical Sciences, Lucknow, IND; 4 Radiodiagnosis, Dr. Ram Manohar Lohia Institute of Medical Sciences, Lucknow, IND

**Keywords:** carpal tunnel release, carpal tunnel syndrome, corticosteroid injection, median nerve, nerve conduction study, neuroparesthesia, nocturnal pain, ultrasound

## Abstract

Background: Carpal tunnel syndrome (CTS) is one of the most common compressive neuropathies. Corticosteroid injection is a pharmacological treatment modality, whereas carpal tunnel release is a surgical treatment option for the management of CTS. Conservative non-pharmacological interventions include wrist splinting, therapeutic exercises, activity modification, ergonomic measures, occupational therapy, and physical therapy. However, the comparative efficacy of corticosteroid injection and surgical decompression remains controversial, particularly when evaluated using objective outcome measures. The present study compared pain relief and median nerve (MN) dynamic mobility following corticosteroid injection and carpal tunnel release in patients with CTS.

Methods: This prospective comparative interventional study included 60 patients with CTS refractory to conservative therapy. Patients were allocated to two groups by alternate consecutive assignment: surgical decompression (Group A, *n* = 30) and corticosteroid injection (Group B, *n* = 30). As this was a prospective comparative interventional study, randomization was not performed. Outcomes were assessed using the Visual Analog Scale (VAS), the Boston Carpal Tunnel Questionnaire (BCTQ), ultrasonographic parameters, and nerve conduction studies over a six-month follow-up period (post-procedure, one week, one month, three months, and six months). MN mobility, cross-sectional area (CSA), nerve conduction velocity, and median distal motor latency were also evaluated. Data were analyzed using IBM SPSS Statistics for Windows, Version 25.0 (Released 2017; IBM Corp., Armonk, NY, USA).

Results: Both groups demonstrated significant improvements in pain and symptom severity. No significant differences were observed between the groups in VAS pain scores or BCTQ Symptom Severity Scale (BCTQ-SSS) scores. At baseline, Group A had mean VAS scores for pain, neuroparesthesia, and functional impairment of 6.43 ± 0.97, 4.43 ± 1.76, and 4.40 ± 1.71, respectively, compared with 5.97 ± 0.93, 4.90 ± 1.65, and 3.63 ± 1.25 in Group B. At six months, these scores decreased to 1.93 ± 0.87, 1.17 ± 0.38, and 1.13 ± 0.35 in Group A and to 1.87 ± 0.68, 1.77 ± 0.97, and 2.27 ± 1.17 in Group B. Mean BCTQ symptom severity scores decreased from 45.03 ± 4.73 to 11.30 ± 2.37 in Group A and from 43.20 ± 6.03 to 11.53 ± 2.15 in Group B. Functional outcomes were significantly better in the surgical group. MN mobility improved significantly following surgery compared with corticosteroid injection. Comparison of MN parameters immediately after the procedure and at six months showed no significant differences between the groups except for MN mobility, which was greater in Group A than in Group B (14.51 ± 1.49 vs. 13.62 ± 0.98; p = 0.008). No significant between-group differences were observed in electrophysiological parameters.

Conclusion: Both treatment modalities were effective in improving symptoms of CTS. However, surgical decompression provided superior functional recovery and greater improvement in MN mobility than corticosteroid injection.

## Introduction

Carpal tunnel syndrome (CTS) is one of the most common compressive neuropathies, resulting from compression of the median nerve (MN) at the wrist. Its prevalence is estimated to be approximately 4% in the general population, with a marked female predominance. Previous studies have reported that females have a four- to fivefold higher risk of developing CTS than males [1,2]. CTS typically presents with pain, numbness, tingling, and weakness involving the thumb, index finger, middle finger, and radial half of the ring finger, often leading to diminished grip strength and impaired hand function [3-6]. These symptoms can significantly affect activities of daily living and occupational performance, frequently necessitating temporary job modifications, such as reducing repetitive hand activities, implementing ergonomic adjustments, or restricting manual tasks. In severe cases, CTS may result in prolonged work absence or even job loss [6,7].

The diagnosis of CTS was established by an experienced orthopedic surgeon based on a combination of characteristic clinical symptoms; physical examination findings, including positive Phalen's and Tinel's tests; and electrodiagnostic confirmation according to accepted clinical guidelines. Treatment options for CTS include conservative non-pharmacological interventions, such as wrist splinting, therapeutic ultrasound, and occupational therapy; pharmacological therapies, including corticosteroid injections, nonsteroidal anti-inflammatory drugs (NSAIDs), diuretics, and vitamin B6 or B12 supplementation; and surgical approaches, including endoscopic carpal tunnel release, open carpal tunnel release, and modified open techniques [8-13].

The choice between corticosteroid injection and surgical decompression depends on several factors, including symptom severity and duration, functional impairment, electrophysiological findings, patient preference, occupational demands, and associated comorbidities. Previous comparative studies have reported inconsistent findings, with some demonstrating comparable short-term outcomes, whereas others have shown superior long-term results following surgical decompression [14-18].

Corticosteroid injection is an established component of conservative management, whereas surgical decompression is generally reserved for patients with persistent or recurrent symptoms despite conservative treatment. Although current treatment algorithms are well established, comparative evaluation of these treatment modalities remains clinically important. Most previous studies have relied primarily on subjective outcome measures, such as pain relief and functional improvement. However, advances in musculoskeletal ultrasonography have enabled the assessment of objective parameters, including MN cross-sectional area (CSA) and nerve mobility, which may provide additional insights into treatment response [12-15].

Therefore, the present study was designed to compare pain relief and dynamic MN mobility following corticosteroid injection and open carpal tunnel release in patients with CTS.

## Materials and methods

Study design

After obtaining approval from the Institutional Ethics Committee (IEC No. 109/23), the study was registered with the Clinical Trials Registry of India (CTRI/2024/09/073211). This prospective, comparative, interventional study was conducted in the Pain Medicine Unit of the Department of Anesthesiology at a tertiary-care teaching and research institute over an 18-month period. A total of 60 patients with CTS who met the predefined inclusion and exclusion criteria and had persistent symptoms despite at least six months of conservative management were enrolled. Conservative management included wrist splinting, activity modification, analgesics or nonsteroidal anti-inflammatory drugs (NSAIDs), and physiotherapy. Patients who had previously undergone ultrasound-guided carpal tunnel release, or open carpal tunnel release, received a corticosteroid injection within the preceding six months, undergone platelet-rich plasma therapy, or received physical therapy within the previous six weeks were excluded. Patients with imaging-confirmed flexor retinaculum rupture, recent use of NSAIDs within one week, thrombocytopenia or other bleeding disorders, vascular insufficiency (defined as an impalpable radial pulse), or peripheral neuropathies other than CTS were also excluded.

Sample size

The sample size was calculated based on the findings of a previous study. Assuming a standardized mean difference of 0.3 and a standard deviation of 0.4 between the two treatment groups, the minimum required sample size to achieve a 95% confidence level and 80% statistical power was calculated using the following formula: 

$n=\frac{2\sigma^{2} \left(Z_{&alpha;/2} + Z_{&beta;} \right)^{2}}{d^{2}}$

where n is the sample size per group, σ is the standard deviation (0.4), Zα/2 is the standard normal deviate corresponding to the 95% confidence level (1.96), Zβ corresponds to 80% power (0.842), and d is the expected difference (0.3) between the two treatment groups.

$n=\frac{2\left( 0.4 \right)^{2}\left( 1.96+0.842 \right)^{2}}{\left( 0.3 \right)^{2}}$

$n=\frac{2\times 0.16\times \left( 2.802 \right)^{2}}{0.09}$

$n=\frac{2\times 0.16\times 7.851}{0.09}$

$n=\frac{2.5123}{0.09}$

*n=27.91* (approx. 28)

To account for an anticipated 10% dropout rate, the calculated sample size was increased from 28 to approximately 31 participants (28 + 2.8 = 30.8). For feasibility, a final sample size of 30 patients per treatment group was selected.

A total of 60 eligible patients with CTS were allocated to one of two treatment groups by alternate consecutive assignment. Allocation was performed without allocation concealment. Group A (n = 30) underwent open carpal tunnel release, whereas Group B (n = 30) received a corticosteroid injection. Randomization was not performed. Owing to the nature of the interventions, neither the participants nor the treating clinicians were blinded to treatment allocation. Outcome assessments were conducted according to a predefined standardized follow-up protocol; however, the outcome assessors were not blinded to treatment allocation.

Procedures

All patients were followed up in the outpatient clinic and underwent clinical assessment using the Visual Analog Scale (VAS) for pain and the Boston Carpal Tunnel Questionnaire (BCTQ), including the Symptom Severity Scale (SSS) and Functional Status Scale (FSS), at baseline (before the intervention) and at one week, one month, three months, and six months after the intervention. MN mobility and CSA were evaluated using ultrasonography at baseline and at three and six months after the intervention. All ultrasonographic assessments were performed by the same consultant pain physician, who was trained in musculoskeletal ultrasonography, using a standardized imaging protocol to minimize interobserver variability. MN conduction velocity and distal motor latency were assessed at baseline and six months after the intervention.

Visual Analogue Scale

Pain intensity was assessed using a 10-cm VAS, where 0 represented "no pain" and 10 represented the "worst imaginable pain." Pain was evaluated at baseline (before the intervention) and at one week, one month, three months, and six months after the intervention.

Boston Carpal Tunnel Questionnaire

Neuropathic symptoms and functional status were assessed using the BCTQ at the same follow-up intervals. The BCTQ, originally described by Levine et al. [11], is a validated patient-reported outcome measure comprising two subscales: the SSS and the FSS. The SSS consists of 11 items, each scored from 1 (mildest symptoms) to 5 (most severe symptoms), with the overall SSS score calculated as the mean of all 11 item scores. The FSS consists of eight items that assess difficulty in performing activities of daily living, each scored from 1 (no difficulty) to 5 (unable to perform the activity), with the overall FSS score calculated as the mean of all eight item scores [11].

Median Nerve Cross-Sectional Area

**Figure 1:**
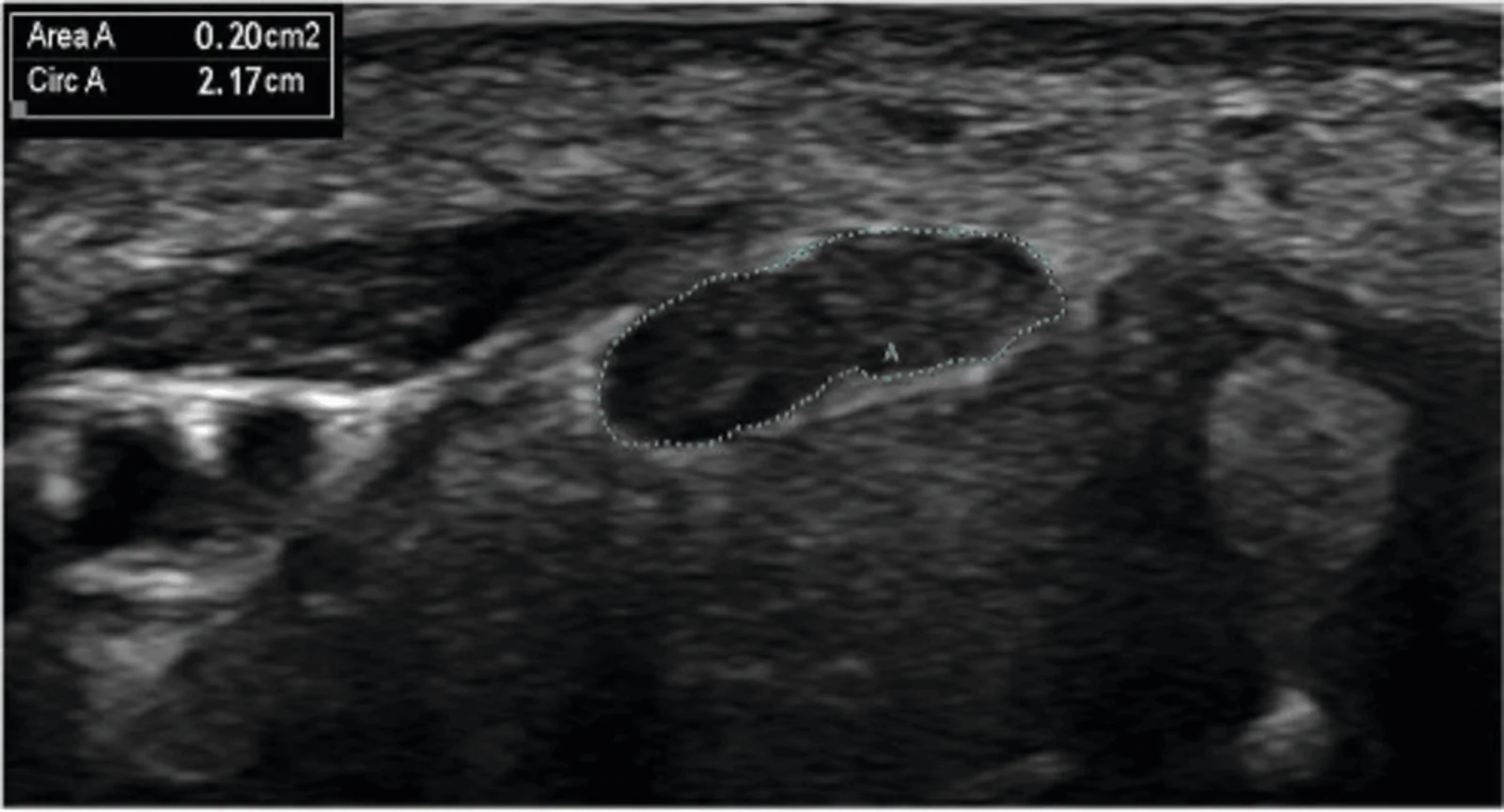
Ultrasonographic measurement of the median nerve cross-sectional area (CSA) at the carpal tunnel inlet. The dotted outline represents manual tracing of the median nerve for CSA measurement, an established parameter for evaluating carpal tunnel syndrome.

The MN CSA was measured statically at the level of the carpal tunnel inlet. All ultrasonographic examinations were performed by a consultant pain physician trained in musculoskeletal ultrasonography using a Konica Minolta Edan Acclarix AX3 ultrasound system (Edan Instruments, Inc., Shenzhen, Guangdong, China) equipped with a 5-17 MHz high-frequency linear-array transducer. Patients were examined in the supine position with the forearm supinated and the wrist maintained in a neutral position. The transducer was placed gently over the MN without applying excessive pressure to avoid deformation of the nerve. The CSA was measured by continuously tracing the inner border of the hyperechoic epineurial rim using the built-in software of the ultrasound system. Three consecutive measurements were obtained, and the mean value was used for statistical analysis [19] (Figure 1).

Median Nerve Mobility

**Figure 2:**
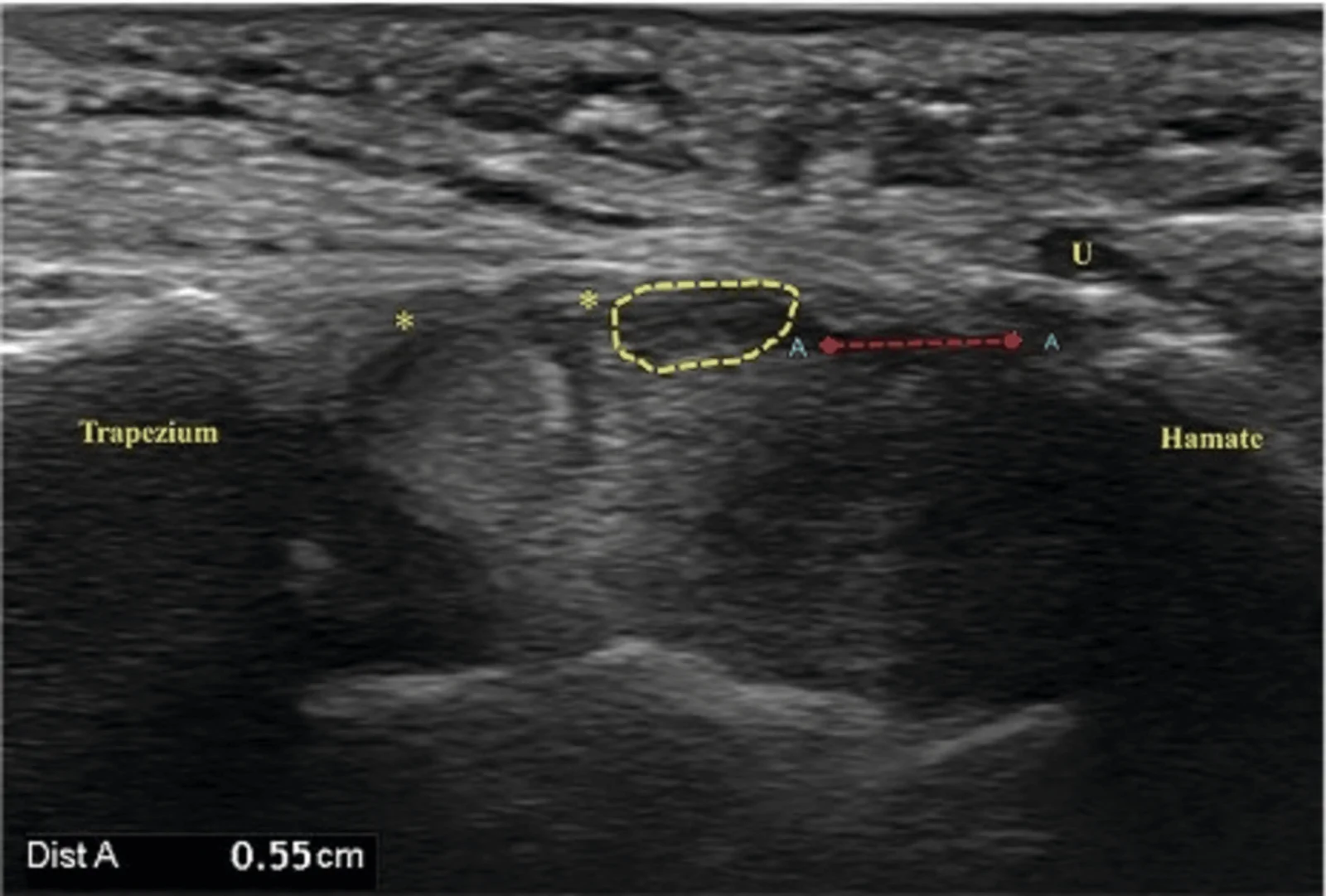
Ultrasonographic assessment of median nerve mobility at the carpal tunnel inlet. The yellow dashed outline indicates the median nerve, and the red dotted line represents the measured distance from the pisiform bone to the median nerve with the wrist in a neutral position.

MN mobility was measured using the method described by Kuo et al. [20]. All ultrasonographic examinations were performed by the same consultant pain physician trained in musculoskeletal ultrasonography. Participants were examined in the supine position with the forearm supinated and resting on the examination table, while the wrist was maintained in a neutral position. The ultrasound transducer was gently placed at the carpal tunnel inlet without applying additional pressure, and the angle of incidence was adjusted to be perpendicular to the MN. After appropriate positioning, participants were instructed to fully flex their fingers into a clenched-fist posture from the initial neutral position and then return to the neutral position. The flexion-extension cycle was completed within three seconds, and a dynamic ultrasound video capturing MN displacement was acquired for subsequent analysis. The measurements were repeated three times, and the mean value was used for statistical analysis (Figure 2).

MN conduction velocity and distal motor latency were assessed using standard nerve conduction studies, whereas ultrasonography was performed separately to evaluate MN morphology and mobility.

Group A (carpal tunnel release)

Open carpal tunnel release was performed by an experienced hand orthopedic surgeon in a well-equipped operating theater using Green's classic approach under local anesthesia. A curvilinear incision approximately 3 cm in length was made 6 mm ulnar to the thenar crease, in line with the longitudinal axis of the fourth finger. Subsequently, approximately 2 cm of the antebrachial fascia, transverse carpal ligament, and palmar fascia between the thenar and hypothenar muscles were incised. The carpal tunnel was then inspected for any abnormalities, including ganglion cysts. External neurolysis of the MN was performed only when the nerve was found to be adherent to the radial leaf of the transverse carpal ligament. Patients were discharged after two hours of observation with instructions to use tramadol 100 mg as rescue analgesia only if required. At the one-week follow-up visit, the splint was temporarily removed for clinical evaluation, including VAS and BCTQ assessments, and was subsequently reapplied as indicated [18].

Group B (local corticosteroid group)

**Figure 3:**
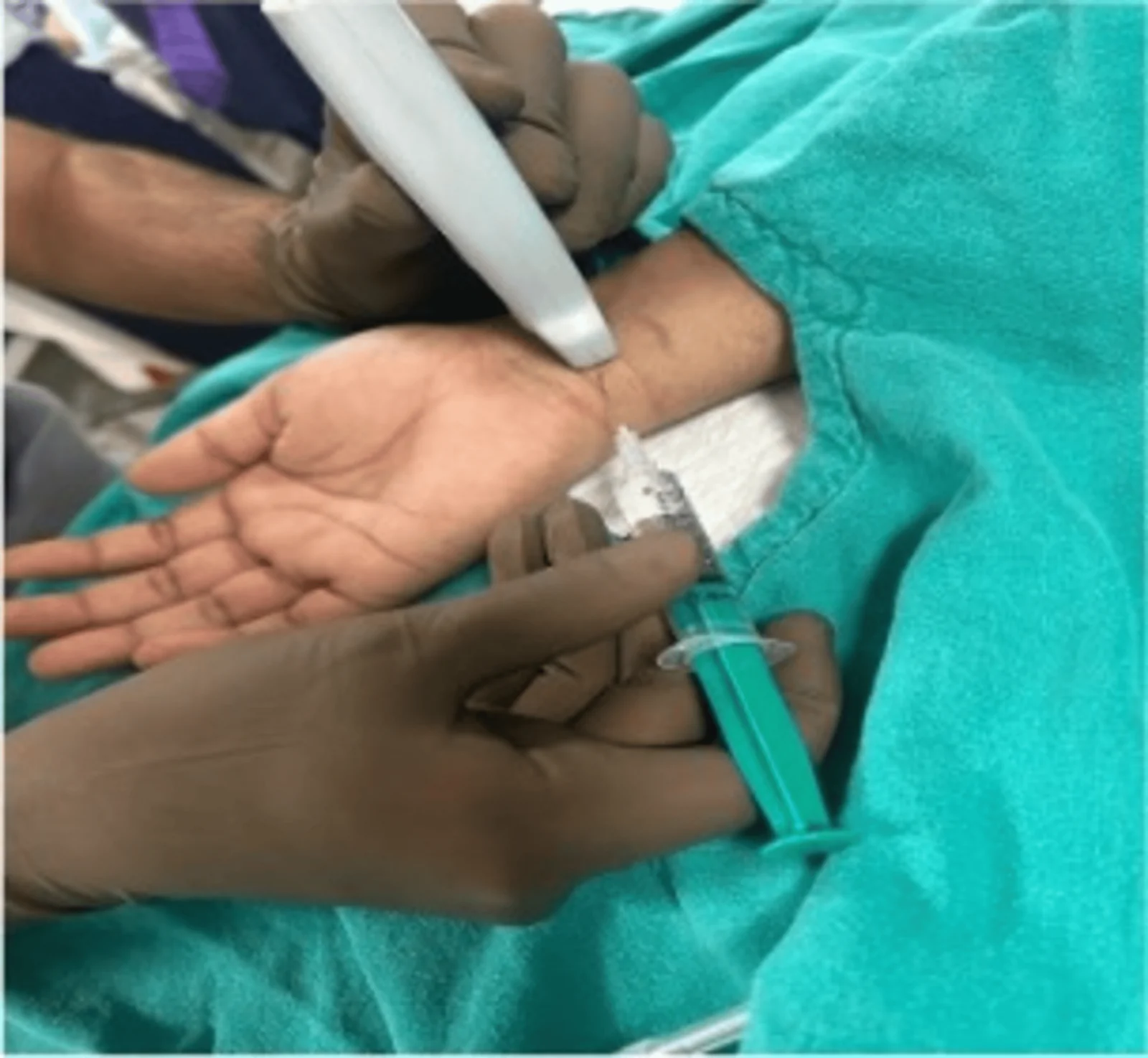
Ultrasound-guided in-plane needle placement after identification of the ulnar artery using color Doppler.

**Figure 4:**
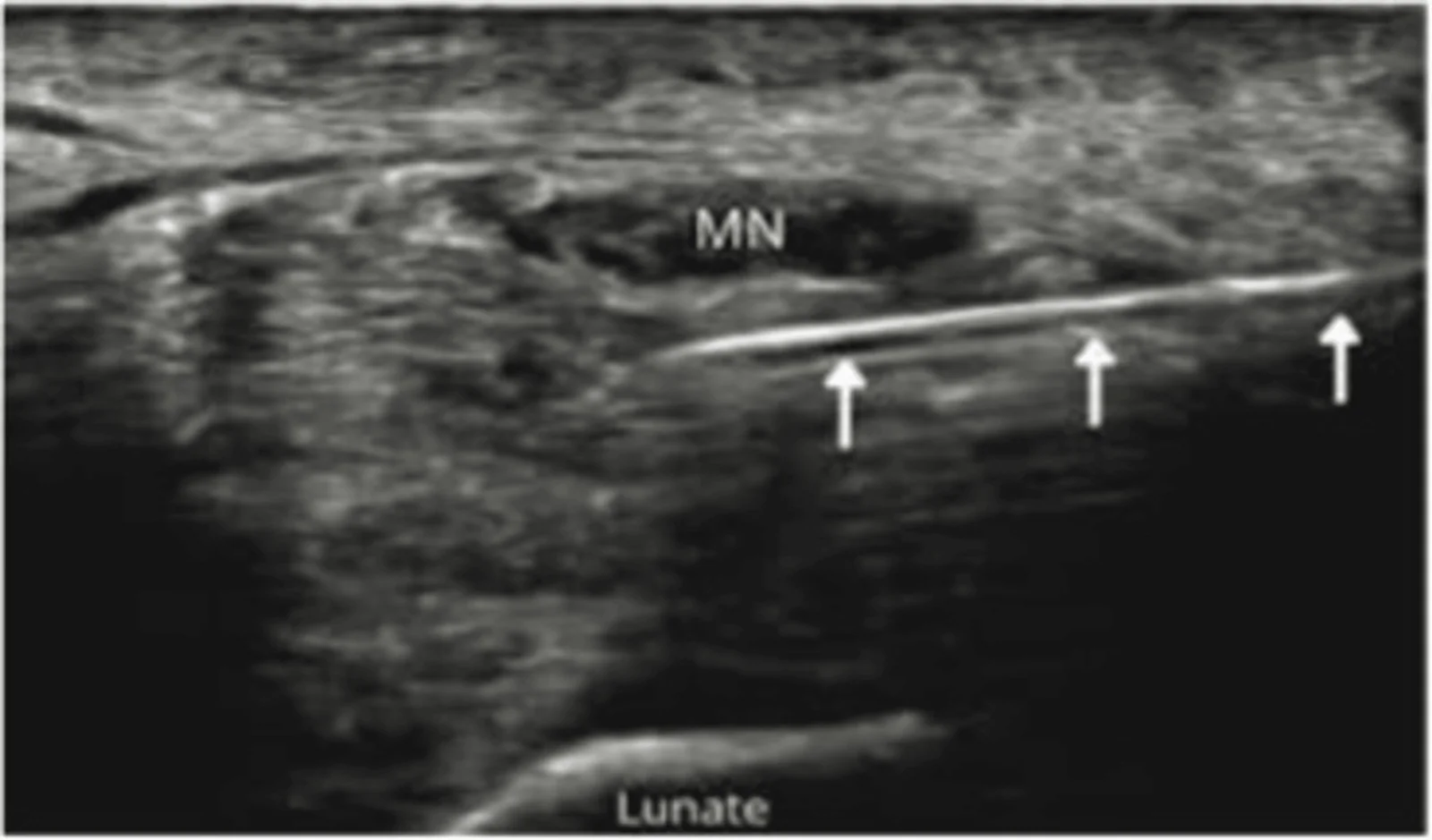
Ultrasound-guided corticosteroid injection into the carpal tunnel. The arrow indicates the needle, and MN denotes the median nerve. The procedure was performed under aseptic conditions using an in-plane ultrasound-guided technique.

Ultrasound-guided corticosteroid injection for CTS was performed by an experienced pain physician in a dedicated minimally invasive pain and spine intervention (MIPSI) laboratory using a Konica Minolta Edan Acclarix AX3 ultrasound system equipped with a 5-17 MHz high-frequency linear-array transducer. The patient's forearm was positioned on a padded bedside table with the fingers slightly flexed to relax the flexor tendons. The transducer was placed transversely over the distal wrist crease. The MN was identified as a hyperechoic bundle of nerve fibers beneath the flexor retinaculum. After aseptic preparation and infiltration with 1% lidocaine for local anesthesia, the ulnar artery was identified and confirmed using color Doppler ultrasonography. Ultrasound-guided hydrodissection of the MN was then performed using an in-plane approach with a 22-gauge, 50-mm needle inserted from the ulnar side. Subsequently, 1 mL of triamcinolone acetonide (40 mg/mL) mixed with 1 mL of 1% lidocaine was injected under ultrasound guidance (Figures 3, 4). Patients were discharged after two hours of observation with instructions to rest for two days, apply cold fomentation, and use tramadol 100 mg as rescue analgesia only if required.

Statistical analysis

Data were collected using a semistructured proforma and subsequently entered into Microsoft Excel 2023 (Microsoft Corp., Redmond, WA, USA) for digitization and data management. Statistical analysis was performed using IBM SPSS Statistics for Windows, Version 25.0 (Released 2017; IBM Corp., Armonk, NY, USA). Categorical variables were expressed as frequencies and percentages, whereas continuous variables were expressed as mean ± standard deviation (SD). The chi-square test and the independent-samples t-test were used to compare categorical and continuous variables, respectively. A two-sided p-value < 0.05 was considered statistically significant.

## Results

Sixty patients who met the predefined inclusion and exclusion criteria were enrolled in the study and allocated to two groups of 30 patients each via alternate-consecutive assignment. The mean age of patients in Groups A and B was 44.93 ± 11.26 years and 42.00 ± 11.99 years, respectively. Females comprised 22 of 30 patients (73.3%) in Group A and 17 of 30 patients (56.7%) in Group B, demonstrating a female predominance in both groups. In Group A, right- and left-sided involvement occurred with equal frequency, whereas in Group B, right-sided involvement was more common than left-sided involvement (18/30 (60.0%) vs. 12/30 (40.0%)). The majority of patients in both groups (17/30 (56.7%) in each group) had a symptom duration of <12 months. There were no statistically significant differences between the two groups with respect to age, sex, side involved, or duration of symptoms (p > 0.05) (Table 1).

**Table 1:** Comparison of age, sex, and baseline clinical profile of patients in the two study groups. CTR: carpal tunnel release.

Characteristic	Group A (CTR) (n = 30)	Group B (corticosteroid) (n = 30)	Statistical significance
				Mean age ± SD (range) years	44.93 ± 11.26 (26-62)	42.00 ± 11.99 (18-60)	t = 0.977, p = 0.333
Sex			χ^2^ = 1.832, p = 0.176, df = 1, Cramer’s V = 0.175
Male	8 (26.7%)	13 (43.3%)	
Female	22 (73.3%)	17 (56.7%)	
Side involved			χ^2 ^= 0.606, p = 0.436, df = 1, Cramer’s V = 0.101
Left	15 (50.0%)	12 (40.0%)	
Right	15 (50.0%)	18 (60.0%)	
Duration of symptoms			χ^2 ^= 0.518, p = 0.972, df = 4, Cramer’s V = 0.093
≤6 months	9 (30.0%)	8 (26.7%)	
>6-12 months	8 (26.7%)	9 (30.0%)	
>12-24 months	7 (23.3%)	8 (26.7%)	
>24-60 months	4 (13.3%)	4 (13.3%)	
>60 months	2 (6.7%)	1 (3.3%)	

Mean VAS scores for pain, neuroparesthesia, and functional impairment at baseline were 6.43 ± 0.97, 4.43 ± 1.76, and 4.40 ± 1.71, respectively, in Group A, compared with 5.97 ± 0.93, 4.90 ± 1.65, and 3.63 ± 1.25, respectively, in Group B. At the six-month follow-up, the corresponding mean VAS scores were 1.93 ± 0.87, 1.17 ± 0.38, and 1.13 ± 0.35 in Group A and 1.87 ± 0.68, 1.77 ± 0.97, and 2.27 ± 1.17 in Group B. There were no statistically significant differences between the two groups in any of the three VAS scores at baseline (p > 0.05). However, at the six-month follow-up, the mean VAS scores for neuroparesthesia and functional impairment were significantly lower in Group A than in Group B (p < 0.05). Overall, the mean absolute and percentage reductions in VAS scores for pain and neuroparesthesia did not differ significantly between the two groups (p > 0.05). In contrast, both the mean absolute and percentage reductions in the VAS score for functional impairment were significantly greater in Group A than in Group B (p < 0.001) (Table 2).

**Table 2:** Comparison of VAS functional score at baseline and different follow-up intervals. CTR: carpal tunnel release, VAS: Visual Analog Scale.

Time interval	Group A (CTR) (n = 30)		Group B (corticosteroid) (n = 30)		Statistical significance
	Mean	SD	Mean	SD	t
Baseline	4.4	1.71	3.63	1.25	1.982
Post-intervention	4.6	1.69	3.8	1.49	1.94
One-week post-intervention	1.33	0.55	2.53	1.28	-4.724
One-month post-intervention	1.2	0.41	2.33	1.18	-4.958
Three-month post-intervention	1.17	0.38	2.47	1.31	-5.236
Six-month post-intervention	1.13	0.35	2.27	1.17	-5.078
Overall reduction in VAS	3.27	1.66	1.37	1.3	4.937
% reduction in VAS	70.17	13.14	33.39	33.59	5.586

Mean BCTQ SSS scores at baseline and at the six-month follow-up were 45.03 ± 4.73 and 11.30 ± 2.37, respectively, in Group A, and 43.20 ± 6.03 and 11.53 ± 2.15, respectively, in Group B. There were no statistically significant differences between the two groups at either time point. Similarly, no statistically significant differences were observed between the groups in the absolute or percentage reductions in symptom severity scores (p > 0.05). Mean BCTQ FSS scores at baseline and at the six-month follow-up were 28.17 ± 4.24 and 9.40 ± 2.98, respectively, in Group A, and 27.97 ± 5.04 and 12.17 ± 2.25, respectively, in Group B. A statistically significant difference between the two groups was observed at the six-month follow-up. Both the absolute and percentage reductions in functional status scores were significantly greater in Group A than in Group B (p < 0.05) (Table 3).

**Table 3:** Comparison of functional severity score (FSS) at baseline and different follow-up intervals. CTR: carpal tunnel release.

Time interval	Group A (CTR) (n = 30)		Group B (corticosteroid) (n = 30)		Statistical significance	
	Mean	SD	Mean	SD	t	p-value
Baseline	28.17	4.24	27.97	5.04	0.166	0.868
Post-intervention	28.1	4.63	28.8	4.44	-0.598	0.552
One-week post-intervention	21.17	4.4	19.87	2.1	1.462	0.149
One-month post-intervention	15.73	3.51	16.4	2.37	-0.861	0.393
Three-month post-intervention	12.1	3.45	14.3	2.37	-2.882	0.006
Six-month post-intervention	9.4	2.98	12.17	2.25	-4.063	<0.001
Overall reduction in FSS	18.77	5.21	15.8	5.9	2.064	0.044
% reduction in FSS	65.81	12.47	54.57	14.76	3.186	0.002

Comparison of MN parameters immediately after the intervention and at the six-month follow-up showed no statistically significant differences between the two groups, except for MN mobility, which was significantly greater in Group A than in Group B (14.51 ± 1.49 vs. 13.62 ± 0.98; p = 0.008) (Table 4).

**Table 4:** Comparison of median nerve mobility. CTR: carpal tunnel release.

Time interval	Group A (CTR) (n = 30)		Group B (corticosteroid) (n = 30)	
	Mean	SD	Mean	SD
Baseline	10.47	0.96	10.76	1.1
Three months	14.26	1.32	13.42	0.96
Six months	14.51	1.49	13.62	0.98

No serious adverse events were observed in either group. During the postoperative period, 6 of 30 patients (20.0%) in Group A experienced pillar pain at the incision site that persisted for more than three months but resolved by the six-month follow-up. In Group B, 3 of 30 patients (10.0%) reported pain at the injection site, which resolved within one week.

## Discussion

The present study demonstrated that corticosteroid injection and carpal tunnel release produced comparable improvements in pain, symptom severity, and most MN parameters. However, carpal tunnel release resulted in superior improvements in neuroparesthesia, functional outcomes, and MN mobility at the final follow-up. Overall, these findings suggest that surgical decompression provides greater functional recovery than corticosteroid injection at six months. These differences may be explained by the distinct mechanisms of action of the two treatment modalities. Corticosteroids primarily exert anti-inflammatory effects, whereas surgical decompression addresses the underlying mechanical compression of the MN, resulting in more sustained functional improvement. Ashworth et al., in a Cochrane review, concluded that surgical decompression resulted in greater improvement in neurophysiological parameters, including MN conduction studies, than conservative treatment [21]. Our findings are generally consistent with this review, as the surgical decompression group demonstrated greater improvements in MN conduction velocity and distal motor latency than the corticosteroid injection group, although these differences were not statistically significant. Similarly, La Costa et al., in a separate meta-analysis, reported that corticosteroid injections provided superior short-term symptom relief, whereas surgical decompression produced better long-term clinical outcomes [22]. 

In the present study, the surgical decompression group demonstrated greater improvement in functional outcomes than the corticosteroid injection group, whereas symptom severity improved similarly in both groups. In contrast, Jafari et al. reported no significant differences between the two groups with respect to either symptom severity or functional outcomes [18]. One possible explanation for this discrepancy is that their study included a greater proportion of patients with mild baseline functional impairment, whereas our study predominantly enrolled patients with moderate functional impairment.

Conversely, Abedi et al. reported greater improvements in both symptom severity and functional outcomes in the surgical decompression group than in the corticosteroid injection group at the six-month follow-up [23]. In our study, however, superior improvement was observed only in functional outcomes. Similarly, Zhang et al. reported improvements in both symptom severity and functional outcomes favoring surgical decompression over corticosteroid injection when treatment-by-time interactions were considered. However, the between-group differences were not statistically significant when evaluated at the overall group level [17]. Likewise, we did not observe significant differences in symptom severity between the two groups. In contrast, Shazad et al. reported greater improvement in symptom scores in the surgical decompression group than in the corticosteroid injection group at all follow-up intervals (3, 6, and 12 months) [24]. Given that surgical decompression addresses the underlying mechanical compression, whereas corticosteroid injection primarily provides symptomatic relief, greater long-term improvement following surgery is biologically plausible. 

Overall, our findings indicate that surgical decompression offers advantages over corticosteroid injection with respect to neuroparesthesia and functional recovery. Similar findings were reported by Akhtar and Khan, who also concluded that surgical decompression was more effective than corticosteroid injection, although they did not clearly describe the criteria used to evaluate treatment efficacy [25].

In addition to pain, symptom severity, and functional outcomes, we evaluated MN mobility, CSA, nerve conduction velocity, and distal motor latency. A significant between-group difference was observed only for MN mobility, which was greater in the surgical decompression group at both the three- and six-month follow-up assessments. Few previous studies have evaluated these objective parameters. Zhang et al. assessed MN cross-CSA, distal motor latency (DML), compound muscle action potential (CMAP), sensory nerve action potential (SNAP), and sensory nerve conduction velocity (SNCV). They reported significant between-group differences at the 12-week follow-up for DML, CMAP, SNCV, and CSA [17]. However, no significant differences were observed at the six-month follow-up. Their study differed from ours because they compared corticosteroid injection alone with combined treatment consisting of corticosteroid injection plus surgical decompression. Therefore, the greater improvements observed during follow-up may have reflected the additive effects of combined therapy. In contrast, Lo et al. reported minimal changes in MN mobility following either treatment and found no significant between-group differences [19]. Likewise, Ashworth et al. concluded that surgical decompression was superior to corticosteroid injection for improving neurophysiological outcomes; however, this conclusion was supported by low-certainty evidence [21]. Given the limited number of studies evaluating objective neurophysiological and ultrasonographic outcomes, further well-designed prospective studies are warranted to provide more robust evidence.

During the postoperative period, approximately 20% of patients who underwent surgical decompression experienced pillar pain at the incision site, which resolved by the six-month follow-up. In the corticosteroid injection group, 10% of patients reported pain at the injection site, which resolved within one week [26].

The present study has several limitations. First, its non-randomized design may have introduced selection bias. Second, the relatively small sample size may have limited the statistical power to detect modest between-group differences. Therefore, larger randomized controlled trials are warranted to validate these findings. In addition, future studies incorporating objective neurophysiological and ultrasonographic assessments may provide further insights into treatment response and recovery.

## Conclusions

Both corticosteroid injection and carpal tunnel release were effective and produced comparable improvements in pain, symptom severity, and most MN parameters. However, carpal tunnel release was associated with greater improvements in functional outcomes and MN mobility. Overall, these findings suggest that corticosteroid injection is an effective option for symptom control, whereas surgical decompression may provide more comprehensive and sustained functional recovery by addressing the underlying mechanical compression of the MN. Corticosteroid injection may be considered for patients with mild-to-moderate disease severity, whereas surgical decompression may be more appropriate for patients with moderate to severe disease or for those who do not respond adequately to conservative or minimally invasive interventions.

Further prospective randomized studies with larger sample sizes and longer follow-up periods are warranted to validate these findings and provide more robust comparative evidence regarding the long-term efficacy of both treatment modalities.
